# Long intergenic non-protein coding RNA 1094 (LINC01094) promotes the progression of breast cancer (BC) by regulating the microRNA-340-5p (miR-340-5p)/E2F transcription factor 3 (E2F3) axis

**DOI:** 10.1080/21655979.2021.1993715

**Published:** 2021-10-28

**Authors:** Xia Wu, Cui Kong, Yilei Wu

**Affiliations:** a Department of Oncology, The Third People’s Hospital of Linyi, Linyi, Shandong, China; b Department of Personnel, The Third People’s Hospital of Linyi, Linyi, Shandong, China; c Department of Both Glandular and Hemangioma Families, Shandong Provincial Third Hospital, Jinan, Shandong, China

**Keywords:** LINC01094, miR-340-5p, E2f3, BC, proliferation, apoptosis

## Abstract

The present study was targeted at investigating the effects of long intergenic non-protein coding RNA 1094 on breast cancer (BC) cell proliferation, apoptosis, and cell cycle and its related mechanism. In this study, Western blot and quantitative real-time polymerase chain reaction (qRT-PCR) were conducted to detect the expressions of LINC01094, microRNA (miRNA, miR)-340-5p, and E2F transcription factor 3 (E2F3) in BC tissues and cells. With transfection, LINC01094 and miR-340-5p expressions were selectively up-regulated or down-regulated in BC cell lines, and then cell proliferation, cell cycle, and apoptosis were examined by cell counting kit-8 (CCK-8), 5-bromo-2ʹ-deoxyuridine (BrdU), and flow cytometry assays. Bioinformatics was utilized to predict the targeted relationships between miR-340-5p and LINC01094, as well as miR-340-5p and E2F3 mRNA 3ʹ-untranslated region (3ʹUTR), and RNA immunoprecipitation (RIP) assay and dual-luciferase reporter gene assay were employed to validate them. It was revealed that, LINC01094 expression was enhanced in BC cells and tissues, and LINC01094 overexpression promoted BC cell proliferation, accelerated cell cycle progression, and inhibited apoptosis while knocking down LINC01094 worked oppositely. LINC01094 directly targeted miR-340-5p and negatively regulated its expression in BC cells. Besides, E2F3 was substantiated to be the target gene of miR-340-5p, and E2F3 expression could be indirectly and positively modulated by LINC01094. All in all, LINC01094 promotes BC cell proliferation and cell cycle progression and inhibits apoptosis via modulating miR-340-5p/E2F3 molecular axis.

## 1. Introduction

Recognized as one of the most common malignancies, breast cancer (BC) poses a serious threat to women’s health [1]. Annually, there is an estimated number of 1.3 million new BC cases worldwide, and over 460,000 patients die due to BC [2]. Surgery, radiotherapy, and chemotherapy are the main treatments of BC [3]. With the advance of treatment techniques, the survival rate of BC patients has been improved significantly, yet some of them still suffer from recurrence and metastasis, which lead to a poor prognosis [4,5]. Nonetheless, the mechanism underlying BC occurrence and development has not been fully deciphered [6].

Long-chain non-coding RNAs (lncRNAs) feature prominently in cancer, and their expression dysregulation contributes to inhibiting or promoting tumor progression [7,8]. Previous studies report that LINC01094 is abnormally expressed and acts as a tumor promoter in several cancers, including ovarian cancer and clear cell renal cell cancer; overexpression of LINC01094 promotes cell proliferation, migration, and invasion [9–11]. In this study, the bioinformatic analysis showed that LINC01094 expression was increased in BC and significantly associated with the poor prognosis of BC patients. Nonetheless, the specific biological functions and mechanism of LINC01094 in BC warrant further investigation.

MicroRNAs (miRNAs, miRs) are small non-coding RNAs with 17–25 nucleotides in length [12]. MiRNAs participate in multiple important biological processes, such as organ development, hematopoiesis, cell proliferation and apoptosis, and tumorigenesis [13]. MiR-340-5p expression is reduced in BC, and miR-340-5p overexpression represses the aggressiveness of BC cells by targeting SOX4 [14]. Besides, it is reported that E2F transcription factor 3 (E2F3) expression is elevated in BC, and E2F3 overexpression facilitates BC cell proliferation and metastasis [15]. In this study, bioinformatics analysis showed that miR-340-5p could target the 3’UTR of E2F3.

In this study, we hypothesized that LINC01094 had the potential to be the diagnostic biomarker and therapeutic target for BC. We detected the functional role and mechanism of LINC01094 in BC progression. Herein, we found that LINC01094 promoted BC cell progression through targeting the miR-340-5p/E2F3 axis.

## 2. Materials and methods

### 2.1 Human tissue sample collection

Fifth-four pairs of BC tissue samples and para-cancerous samples used in this study were all selected from the surgically removed tumorous tissues and matched para-tumorous tissues in The Third People’s Hospital of Linyi and The Shandong Provincial Third Hospital, and then frozen in liquid nitrogen. Before the surgery, none of the participants had undergone radiotherapy, chemotherapy, and other related treatments, and every subject signed the informed consent form. The present study was endorsed by and conducted under the guidance of the hospital’s ethics committee.

### 2.2 Cell culture *[16]*

From ATCC (Manassas, VA, USA), human breast epithelial cell (MCF-10A), and BC cell lines (MCF-7, L6, MDA-MB-231, and DU4475) were bought. MCF10CA1a cell line was obtained from Karmanos Cancer Institute (Detroit, MI, USA). Dulbecco’s modified Eagle medium (DMEM, Invitrogen, Carlsbad, CA, USA) containing 10% fetal bovine serum, 100 μg/ml streptomycin, and 100 U/ml penicillin (Invitrogen, Carlsbad, CA, USA) was used for culturing the cells mentioned above in 5% CO_2_ and 95% humidity at 37°C. The medium was refreshed every 48 h, and the cells were sub-cultured every 96 h.

### 2.3 Cell transfection *[17]*

MDA-MB-231 and MCF-7 cells were transferred at 1 × 10^6^ cells/ml into 60-mm culture plates and cultured at 37°C for 24 h in 5% CO_2_. Then, the transfection was conducted with Lipofectamine® 2000 (Invitrogen, Carlsbad, CA, USA). LINC01094 overexpression plasmid (LINC01094), pcDNA empty vector (NC), small interfering RNAs against LINC01094 (si-LINC01094-1 and si-LINC01094-2) and the negative control (si-NC), miR-340-5p inhibitors/mimics and their controls inhibitors/mimics NC were obtained from RiboBio (Guangzhou, China). After 24 h, quantitative real-time polymerase chain reaction (qRT-PCR) was utilized for measuring the transfection efficiency before the cells were collected for follow-up experiments.

### 2.4 qRT-PCR *[18]*

Total RNA was extracted from tissues or cultured cells with TRIzol reagent (Invitrogen, Shanghai, China). A Reverse Transcription Kit (Takara, Dalian, China) was employed to reversely transcribe RNA into cDNA. Then, a total volume of 25 μL mixture (2× SYBR Premix Eaq^TM^ II (12.5 μL; Takara, Dalian, China), PCR forward primers (1 μL; 10 μmol/L), PCR reverse primers (1 μL; 10 μmol/L), 2 μL of cDNA and ddH_2_O) was prepared for each sample, and the two-step amplification was conducted. The initial denaturation was performed at 95°C for 30 s in 1 cycle; PCR reaction was performed for 5 s at 95°C and for 30 s at 60°C in 40 cycles. The relative expressions of miR-340-5p, LINC01094, and E2F3 were calculated by the 2^−ΔΔCt^ method, with U6 and GAPDH as the internal references. Below are the primer sequences (F: forward; R: reverse).

LINC01094: (F) 5ʹ-TGTAAAACGACGGCCAGT-3ʹ, (reverse) 5ʹ-CAGGAAACAGCTATGACC-3ʹ;

miR-340-5p: (F) 5ʹ-CCGTTAGTTACGATTCGAAG-3ʹ, (R) 5ʹ-AGGCCGCGCGTAGTGATGCAACA-3ʹ;

E2F3: (F) 5ʹ-CCTGGA TACCGCAGCTAGGA-3ʹ, (R) 5ʹ-GCGGCGCAATACGAATGCCCC-3ʹ;

GAPDH: (F) 5ʹ-AAGGTGAAGGTCGGAGTCAAC-3ʹ, (R) 5ʹ-GGGGTCATTGATGGCAACAATA-3ʹ;

U6: (F) 5ʹ-CTCGCTTCGGCAGCACATATACT-3ʹ, (R) 5ʹ-ATTTGCGTGTCATCCTTGCGCA-3ʹ.

### 2.5 Cell counting kit-8 (CCK-8) assay *[19]*

We collected the cells during logarithmic growth in each group and adjusted the cell concentration to 1 × 10^5^ cells/ml. Each well in 96-well plates was added with 100 μL of cell suspension, followed by routine cell culture. At 24, 36, 48, and 72 h, the cells were incubated with 10 μL of CCK-8 solution (Dojindo Molecular Technologies, Japan) for another 2 h. Then, the optical density (OD) at 490 nm of cells in each well was detected by the microplate reader.

### 2.6 5-bromo-2ʹ-deoxyuridine (BrdU) assay *[20]*

MDA-MB-231 and MCF-7 cells were transferred into 24-well plates (with a cover glass in each well, 2.5 × 10^5^ cells/well), respectively. After 24 h, the cells were incubated with BrdU solution (Beyotime Biotechnology, Shanghai, China) for another 4 h and then fixed for 30 min with 4% paraformaldehyde. After the supernatant was discarded, the cells were incubated with anti-BrdU antibody (1: 500, Beyotime Biotechnology, Shanghai, China) for 30 min at room temperature and then with 100 μL of 1× Hoechst 33,342 reaction solution at room temperature in the dark for 20 min, followed by being rinsed with PBS. In 10 random high-power fields under the microscope, the number of BrdU-positive cells and the total number of cells were calculated, and the average was taken.

### 2.7 Flow cytometry *[21]*

The cells rinsed twice with Pre-cooled PBS were re-suspended (200 μL), and then 10 μL of Annexin V-FITC fluorescent probe was added to the cell suspension. After being gently shaken, mixed, and incubated in the dark for 30 min, the cell suspension was incubated with 5 μL of propidium iodide (PI) staining solution for 30 min in the dark and then added with 400 μL of binding buffer. Eventually, the cells were filtered with a 300-mesh nylon net and measured with a flow cytometer.

### 2.8 Dual-luciferase reporter gene assay *[22]*

The binding sites between miR-340-5p and LINC01094, as well as between E2F3 mRNA 3ʹ-untranslated region (3ʹUTR) and miR-340-5p, were predicted by the StarBase database and TargetScan database, respectively. According to the binding site, wild-type E2F3 (WT E2F3), wild-type LINC01094 (WT LINC01094), mutant E2F3 (MUT E2F3), and mutant LINC01094 (MUT LINC01094) dual-luciferase reporter vectors were designed and constructed. The above-mentioned reporter vectors, miR-340-5p mimics, mimics NC and miR-340-5p inhibitors, and inhibitors NC were co-transfected into MDA-MB-231 and MCF-7 cells, respectively. After 48 h, the cells were harvested, lysed, and then centrifuged for 3 min before the supernatant was collected. On a Dual-Luciferase Reporter Assay System (Promega, Madison, WI, USA), the luciferase activity was measured following the manufacturer’s instructions, and the ratio of the luciferase activity of Renilla luciferase to that of firefly luciferase was utilized to show the binding intensity of LINC01094 to miR-340-5p, as well as of E2F3 3ʹUTR with miR-340-5p.

### 2.9 RNA immunoprecipitation (RIP) assay *[23]*

A RIP assay kit (Millipore, Billerica, MA, USA) was employed to validate the binding relationship between LINC01094 and miR-340-5p. Briefly, the cells were lysed by Radio-Immunoprecipitation Assay (RIPA) lysis buffer (Beyotime Biotechnology, Co., Shanghai, China) for 5 min on the ice, and the supernatant was harvested after 10 min of centrifugation at 14,000 rpm and 4°C. A portion of the supernatant was used as input, and the other was incubated with antibodies for co-precipitation. After collecting magnetic bead-protein complex, proteinase K was utilized to detach the input and samples to extract RNA for follow-up PCR detection

### 2.10 Western blot *[24]*

RIPA lysis buffer (Beyotime Biotechnology, Shanghai, China) with protease inhibitors was utilized for cell lysis on the ice for 40 min. The supernatant was harvested after 15 min of centrifugation at 4°C at 10,000 r/min, and the protein concentration was gauged by the BCA method. The protein samples and a 1/5 volume of 5× SDS-PAGE loading buffer were fully mixed and then boiled for 5 min at 100°C. Subsequently, SDS-PAGE gel was prepared, and each well was added with an equal amount of protein sample. Next, the electrophoresis was performed. The proteins were subsequently transferred to PVDF membranes. After blocking the nonspecific antigens by 5% skimmed milk, the membranes were incubated with primary antibody anti-E2F3 antibody (ab152126, 1:1000) at 4°C overnight and then with secondary antibody goat anti-rabbit IgG H&L (HRP) (ab205718, 1:2000) for 1 h at room temperature. At last, the protein bands were developed with hyper-sensitive ECL (Millipore, Bedford, MA, USA). All antibodies were bought from Abcam (Shanghai, China).

### 2.11 Lung metastasis model in vivo

The animal experiments were approved by the Animal Care and Use Committee of The Third People’s Hospital of Linyi. Male BALB/c nude mice (8 weeks old) were used to establish the lung metastasis model. In each group, approximately 5 × 10^6^ MDA-MB-231 cells transfected with LINC01094 overexpression plasmids or control plasmids were injected into the caudal vein of each mouse (10 mice per group). After 3 weeks, the mice were sacrificed, and lung tissues were obtained. Then hematoxylin and eosin (H&E) staining was performed, and the metastatic nodules in the lung tissues were observed and evaluated by a pathologist.

### 2.12 Statistical analysis

All experiments were independently conducted in triplicate. The SPSS 22.0 software was employed to analyze the experimental data, which were expressed as the ‘mean ± standard error’. The data conforming to normal distribution between two groups were analyzed by *t*-test, and the data of multiple experimental groups were analyzed through one-way ANOVA to calculate the *P*-value. *P* < 0.05 implied that the difference was of statistical significance.

## 3. Results

We hypothesized that LINC01094 could promote the progression of BC. Gain-of-function and loss-of-function models were established, and we substantiated that LINC01094 could regulate the malignant biological behaviors of BC cells *in vitro*. Additionally, we found that LINC01094 directly targeted miR-340-5p and up-regulated E2F3 expression, thus promoting BC cells proliferation and inhibiting apoptosis.

### 3.1 LINC01094 expression is up-regulated in BC tissues

**Figure 1.**
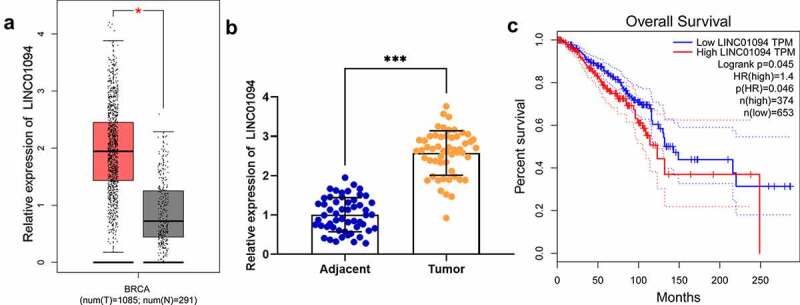
LINC01094 expression is up-regulated in BC tissues

Using the GEPIA database (http://gepia2.cancer-pku.cn/#index) to analyze LINC01094 expression characteristics in BC tissues of TCGA data, we found that LINC01094 expression was increased in BC tissues as against normal tissues (Figure 1a). qRT-PCR showed that LINC01094 expression was markedly higher in BC tissues in comparison to para-cancerous tissues (Figure 1b). Furthermore, high LINC01094 expression was linked to patients’ short overall survival time (Figure 1c).

(a) With the GEPIA database, box plots were adopted for analyzing LINC01094 expression difference in BC and para-tumorous tissues. (b) Detection via qRT-PCR of LINC01094 expression in 54 cases of BC and para-cancerous tissues. (c) According to GEPIA database, the relationship between LINC01094 expression and the BC patient’s survival was analyzed. *P < 0.05, **P < 0.01 and ***P < 0.001.

### 3.2 LINC01094 has effects on BC cell proliferation, cell cycle progression, and apoptosis

**Figure 2.**
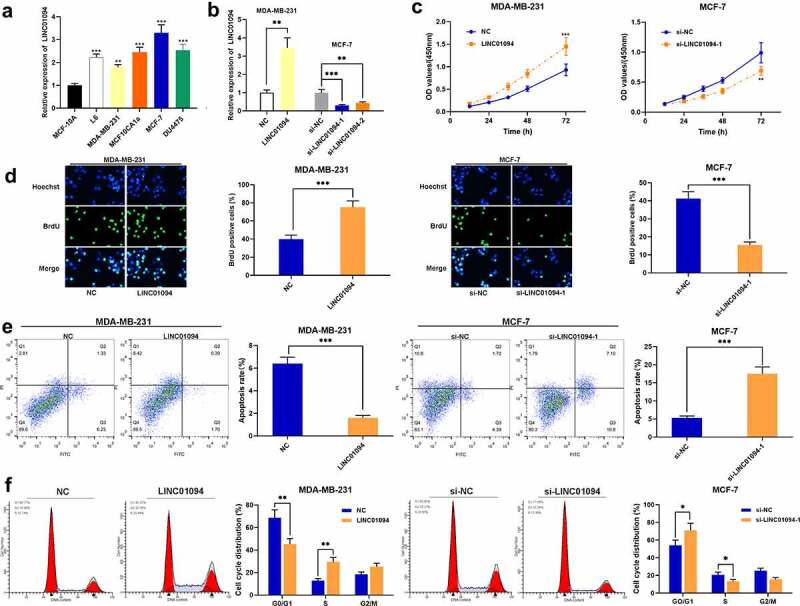
LINC01094 has effects on cell proliferation, cell cycle progression and apoptosis

To elaborate on LINC01094’s biological functions in BC, qRT-PCR was conducted to further quantify LINC01094 expression in human mammary epithelial cells MCF-10A and BC cell lines, and it showed that LINC01094 was highly expression in BC cells compared with that in MCF-10A cells, and it showed that, among BC cell lines, LINC01094 was highly expressed in MCF-7 cells and lowly expressed in MDA-MB-231 cells (Figure 2a). Thus, MDA-MB-231 cells and MCF-7 cells were used for LINC01094 overexpression and knockdown experiments, respectively. LINC01094 overexpression plasmid was transfected to MDA-MB-231 cells, si-LINC01094-1 and si-LINC01094-2 were transfected to MCF-7 cells, and the transfection efficiency was determined via qRT-PCR (Figure 2b). CCK-8, BrdU and flow cytometry assays showed that as against the control group, LINC01094 overexpression markedly promoted BC cell proliferation, suppressed cell apoptosis, and accelerated cell cycle progression, while LINC01094 knockdown significantly reduced BC cell proliferation, blocked the cell cycle in G0/G1 phase, and induced apoptosis (Figure 2c-f).

(a) Detection by qRT-PCR of LINC01094 expression in BC cell lines (MCF-7, L6, MDA-MB-231, MCF10CA1a and DU4475) and MCF-10A cells. (b) Detection via qRT-PCR of the transfection efficiency of LINC01094, si-LINC01094-1 and si-LINC01094-2 in MCF-7 and MDA-MB-231 cells. (c,d) BrdU and CCK-8 assays were performed to detect the effects of LINC01094 on MDA-MB-231 and MCF-7 cells’ proliferation. (e,f) The effects of LINC01094 on MDA-MB-231 and MCF-7 cell apoptosis and cell cycle were detected by flow cytometry assay. *P < 0.05, **P < 0.01 and ***P < 0.001

### 3.3 LINC01094 directly targets miR-340-5p

**Figure 3.**
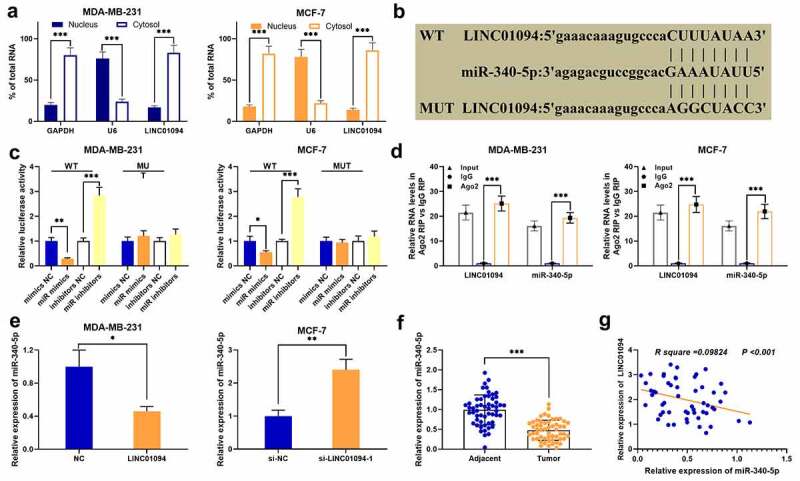
LINC01094 directly targets miR-340-5p in BC cells

To dig deeper into the downstream mechanism of LINC01094, the subcellular localization of LINC01094 was detected by qRT-PCR after the nucleocytoplasmic separation experiment, and it was revealed that LINC01094 was primarily located in the cytoplasm of MCF-7 and MDA-MB-231 cells (Figure 3a). Next, StarBase database was applied to predict its targets, and miR-340-5p was among the predicted target candidates (Figure 3b). Subsequently, the predicted binding site between LINC01094 and miR-340-5p was validated by a dual-luciferase reporter gene assay. It was revealed that the transfection of miR-340-5p mimic could repress WT LINC01094’s luciferase activity, and the transfection of miR-340-5p inhibitors exerted an opposite effect (Figure 3c). Besides, the RIP assay confirmed that in contrast with control IgG group, miR-340-5p and LINC01094 were significantly enriched in Ago2-containing microribonucleoproteins (Figure 3d). Then, we detected miR-340-5p expression in BC cells and tissues via qRT-PCR, and it was revealed that LINC01094 overexpression significantly decreased miR-340-5p expression, and yet knocking down LINC01094 significantly up-regulated miR-340-5p expression (Figure 3e). MiR-340-5p was remarkably down-regulated in BC tissues compared to para-cancerous tissues, and LINC01094 and miR-340-5p expressions were inversely correlated in BC tissues (figure 3f-g). These data suggested that in BC, LINC01094 directly targeted miR-340-5p to repress its expression.

(a) The subcellular location of LINC01094 was assessed via qRT-PCR after nucleocytoplasmic separation. (b) StarBase database predicted the binding site between LIN01094 and miR-340-5p. (c) MCF-7 and MDA-MB-231 cells were co-transfected with MUT LINC01094 or WT LINC01094 reporter with miR-340-5p inhibitors or mimics, respectively; the dual-luciferase reporter gene system was utilized to detect the luciferase activity. (d) RIP assay was employed to verify the binding relationship between LINC01094 and miR-340-5p. E. The effects of LINC01094 overexpression and knockdown on miR-340-5p expression in BC cells were detected via qRT-PCR. F. MiR-340-5p expression in 54 cases of BC and para-cancerous tissues was detected via qRT-PCR. G. Pearson correlation analysis of the correlation between LINC01094 and miR-340-5p expressions in BC tissues. *P < 0.05, **P < 0.01 and ***P < 0.001.

### 3.4 LINC01094 up-regulates E2F3 expression through adsorbing miR-340-5p

**Figure 4.**
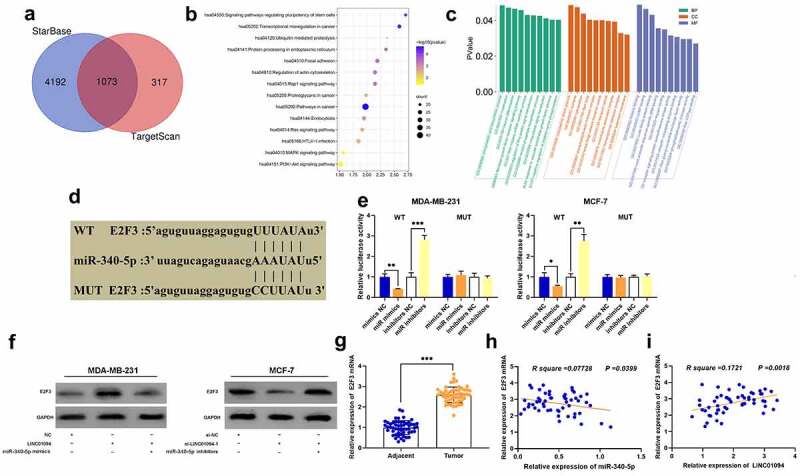
LINC01094 up-regulates E2F3 expression via sponging miR-340-5p

Next, the StarBase database and TargetScan database were utilized to predict the downstream target genes of miR-340-5p, and it was revealed that there were a total of 1073 candidate targets (Figure 4a). Then, the Kyoto Encyclopedia of Genes and Genomes (KEGG) database was applied to analyze the signaling pathways in which the above-mentioned target genes were enriched, and it was indicated that the target genes of miR-340-5p were mainly enriched in the pathways in cancer, PI3K-Akt signaling pathway, and endocytosis, which were closely related to BC progression (Figure 4b). GO enrichment analysis manifested that they were mainly enriched in phosphatidic acid biosynthetic process and histone binding, etc (Figure 4c). Among the potential targets, E2F3 was associated with BC development (Figure 4d). The dual-luciferase reporter gene assay manifested that the transfection of miR-340-5p mimics could repress WT E2F3’s luciferase activity, and the transfection of miR-340-5p inhibitors worked oppositely; no significant effects were observed on MUT E2F3’s luciferase activity (Figure 4e). Western blot suggested that LINC01094 overexpression could up-regulate E2F3 expression whereas the transfection of miR-340-5p mimics could partially counteract this effect; LINC01094 knockdown could down-regulate E2F3 expression whereas the transfection of miR-340-5p inhibitors could partially reverse this effect (figure 4f). Besides, qRT-PCR showed that as against adjacent tissues, E2F3 mRNA was high-expressed in BC tissues, and E2F3 mRNA and miR-340-5p expressions were inversely related in BC tissues whereas LINC01094 and E2F3 mRNA expressions were positively correlated (Figure 4g-i).

(a) StarBase database and TargetScan database were employed for predicting downstream miR-340-5p targets. (b, C) KEGG and GO databases were used to perform a pathway enrichment analysis of the target genes of miR-340-5p. (d) The online database StarBase was applied to predict the binding site of miR-340-5p to E2F3 mRNA 3ʹUTR. E. MUT E2F3 or WT E2F3 luciferase reporter vectors were co-transfected with miR-340-5p inhibitors or mimics into MCF-7 and MDA-MB-231 cells, and then the luciferase activity was detected. (f) Western blot was conducted to detect the regulatory effects of LINC01094 and miR-340-5p on E2F3 expression. (g) E2F3 mRNA expression in 54 pairs of BC tissues and para-cancerous tissues was examined through qRT-PCR. H-I. Pearson correlation analysis of the correlation among LINC01094, E2F3 mRNA and miR-340-5p expressions in BC tissues. *P < 0.05, **P < 0.01 and ***P < 0.001.

### 3.5 LINC01094, miR-340-5p, and E2F3 have effects on BC cell proliferation, cycle, and apoptosis

**Figure 5.**
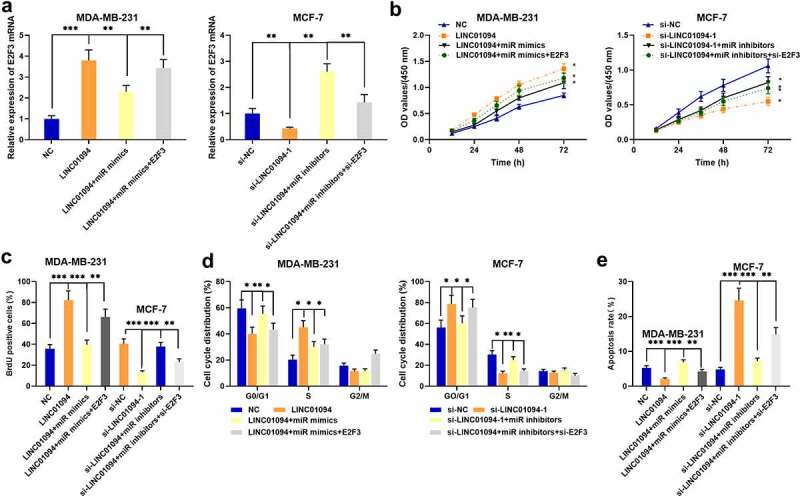
The impacts of LINC01094 and miR-340-5p of BC cell proliferation, cycle progression and apoptosis

To delve deeper into the influences of LINC01094, miR-340-5p and E2F3 on BC cell proliferation, cell cycle and apoptosis, we transfected LINC01094 overexpression plasmids, LINC01094 overexpression plasmids + miR-340-5p mimics and LINC01094 overexpressoin plasmids + miR-340-5p mimics + E2F3 overexpression plasmids into MDA-MB-231 cells, and transfected si-LINC01094-1, si-LINC01094-1 + miR-340-5p inhibitors and si-LINC01094-1 + miR-340-5p inhibitors + si-E2F3 into MCF-7 cells. qRT-PCR showed the transfection was successful (Figure 5a). Then, through CCK-8, BrdU, and flow cytometry assays, we discovered that as opposed to the control group, LINC01094 overexpression observably induced BC cell proliferation, inhibited the apoptosis, and boosted the cell cycle progression, yet the transfection of miR-340-5p weakened these effects, and E2F3 overexpression reversed the anti-proliferative effects of miR-340-5p (Figure 5b-e); LINC01094 knockdown notably suppressed cell proliferation, induced the apoptosis and blocked the cell cycle in G0/G1 phase whereas the transfection of miR-340-5p inhibitors counteracted the above effects, and E2F3 knockdown abolished the proliferative effects of miR-340-5p inhibitors (Figure 5b,e).

A. LINC01094 overexpression plasmids, LINC01094 overexpression plasmids + miR-340-5p mimics and LINC01094 overexpressoin plasmids + miR-340-5p mimics + E2F3 overexpression plasmids were into MDA-MB-231 cells, respectively, and si-LINC01094-1, si-LINC01094-1 + miR-340-5p inhibitors and si-LINC01094-1 + miR-340-5p inhibitors + si-E2F3 were transfected into MCF-7 cells, respectively. The transfection efficiency was measured via qRT-PCR. B-C. BrdU and CCK-8 assays were utilized to detect the impacts of LINC01094, miR-340-5p, E2F3 on BC cell proliferation. D-E. Flow cytometry was utilized for detecting the impacts of LINC01094, miR-340-5p and E2F3 on BC cell apoptosis and cycle progression. *P < 0.05, **P < 0.01 and ***P < 0.001.

### 3.6 Overexpression of LINC01094 promoted the lung metastasis of BC in vivo

**Figure 6.**
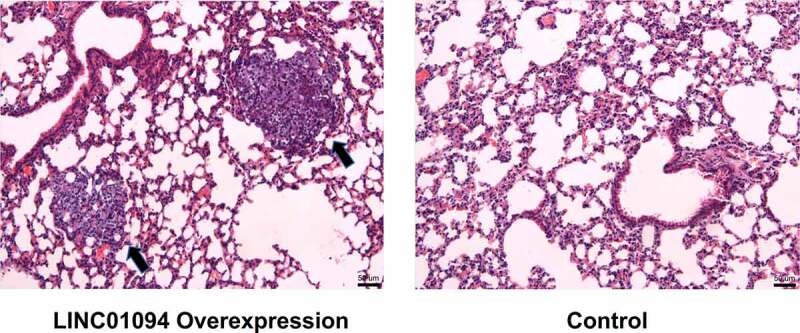
LINC01094 promoted the lung metastasis of MDA-MB-231 cells *in vivo.*

To further validate that LINC01094 could promote BC progression, MDA-MB-231 cells transfected with LINC01094 or control plasmids were respectively injected into the tail vein of the nude mice. H&E staining of lung tissue of the mice showed that, in the control group, metastatic nodules could be detected in 5 of the mice; while in LINC01094 overexpression group, metastatic nodules are detected in all of the 10 mice, and the metastasis was much severer than that in the control group (Figure 6), suggesting that LINC01094 promoted the lung metastasis of BC *in vivo*.

H&E staining was used to detect the metastatic nodule of the mice, which were injected with MDA-MB-231 cells transfected with LINC01094 overexpression plasmids or control plasmids, and the representative images were shown

## 4. Discussion

LncRNAs have the potential to be used as biomarkers and targets for various cancers [25]. Generally, lncRNA dysregulation affects a lot of cellular biological processes, for example, apoptosis, proliferation, movement, migration, angiogenesis, etc [25,26]. For instance, Lnc-BM is abnormally high-expressed in BC and is linked to the adverse prognosis of the patients; Lnc-BM overexpression promotes the expressions of STAT3-dependent ICAM1 and CCL2 and further activates the JAK2/STAT3 pathway, by which it promotes brain metastasis of BC cells [27]. LINC01094 expression is elevated in renal cell cancer tissues and cells, and LINC01094 overexpression down-regulates miR-577 expression and promotes the translation of FOXM1 mRNA, by which it increases the radioresistance of renal cell carcinoma cells [10]. In this work, it was demonstrated that high LINC01094 expression was associated with shorter overall survival time, and the *in vivo* experiments indicated that LINC01094 facilitated BC cells growth. The aforementioned findings suggest that LINC01094 has the potential to be a diagnostic biomarker and treatment target for BC patients. But the mechanism of the dysregulation of LINC01094 expression is not explored in the present work, and the following studies should investigate whether the up-regulation of LINC01094 expression is associated with the aberrant methylation of the promoter or transcriptional activation.

Many miRNAs are associated with the tumorigenesis and progression of malignancies [28]. Their main function is to act on target mRNA to promote its degradation or inhibit its translation, and they have important application prospects in the diagnosis and treatment of malignancies [29]. For example, miR-222 expression is enhanced in BC tissues, and miR-222 overexpression regulates the phenotypes of BC-related fibroblasts to promote the aggressiveness of BC cells [30]. MiR-92a-3p expression is also enhanced in BC tissues and cells, and its high expression is related to increased TNM stage and larger tumor size of BC patients [31]. As the targeted miRNA of LINC00662, miR-340-5p expression is reduced in colonic carcinoma tissues, and the inhibition of miR-340-5p expression promotes colonic carcinoma cell proliferation, migration, and invasion, and restrains apoptosis [32,33]. In BC tissues, miR-340-5p expression is reduced, which is closely associated with the clinicopathological indicators of patients, and inhibiting miR-340-5p expression helps stabilize ZEB2 protein level and promote the metastasis of BC cells [34]. In this study, bioinformatics analysis predicted a binding site between LINC01094 and miR-340-5p, and this prediction was validated by dual-luciferase reporter gene and RIP assays. Furthermore, there was a negative correlation between them, and the *in vitro* experiments indicated that miR-340-3p overexpression reversed the promoting effects of overexpression LINC01094 on BC cell proliferation and apoptosis. These results implied that LINC01094 promoted tumor progression by targeting miR-340-5p.

E2F3 belongs to E2F transcription factor family (E2F1-8), and its gene is located on chromosome 6p22 [35,36]. Previous reports show that the dysregulation of E2F transcription factor is presented in many cancers, including bladder cancer, BC, ovarian cancer, prostate cancer, gastrointestinal cancer, and lung cancer [37–39]. Reportedly, E2F3 accelerates tumor progression both *in vivo* and *in vitr*o, and its expression is increased in different tumor types, such as, lung cancer, prostate cancer, bladder cancer, and retinoblastoma [40–43]. E2F3 is highly expressed in gastric carcinoma tissues and regulates DKK3 expression by modulating miR-125a expression, ultimately promoting gastric cancer cell proliferation, migration, and invasion [44]. E2F3 is a direct downstream target of HOXB9 and is overexpressed in endometrial carcinoma tissues, and E2F3 overexpression enhances endometrial carcinoma cell migration ability [45]. Importantly, E2F3 expression in BC is significantly up-regulated, which is positively related to the survival time of BC patients, and knocking down E2F3 can inhibit BC cell proliferation, migration, and invasion, as well as the formation of 3D sphere [46]. This complies with the results of this study. In addition, this study authenticated that E2F3 was a direct downstream miR-340-5p target. MiR-340-5p and E2F3 mRNA expressions were inversely correlated, and E2F3 mRNA and LINC01094 expressions were positively correlated in BC samples. We also observed that LINC01094 up-regulated E2F3 expression by adsorbing miR-340-5p. Our data help clarify the mechanism of miR-340-5p and E2F3 dysregulation in BC. It is interesting to explore whether LINC01094 can regulate other downstream target genes in the following studies.

## 5. Conclusion

In summary, LINC01094 induces cell cycle progression, promotes BC cell proliferation, and inhibits apoptosis through modulating the miR-340-5p/E2F3 molecular axis. This study may provide some clues for early diagnosis, prognostic judgment, and gene therapy of BC. Nevertheless, there are several shortcomings of this work. Firstly, other target of LINC01094 and miR-340-5p remain to be screened, which will help further clarify the mechanism of BC progression. Additionally, this study is still limited to single-center samples. In the future, we will enlarge tissue samples and explore the prognostic prediction value of LINC01094 in depth.

## Data Availability

The data used to support the findings of this study are available from the corresponding author upon request.

## Research highlights

LINC01094 expression was up-regulated in BC tissues and associated with a poor prognosis.

LINC01094 overexpression significantly promoted BC cell proliferation and inhibited apoptosis.

LINC01094 functioned as the sponge of miR-340-5p to upregulate E2F3 expression.
